# Prediction of Suicide Attempts Using Clinician Assessment, Patient Self-report, and Electronic Health Records

**DOI:** 10.1001/jamanetworkopen.2021.44373

**Published:** 2022-01-27

**Authors:** Matthew K. Nock, Alexander J. Millner, Eric L. Ross, Chris J. Kennedy, Maha Al-Suwaidi, Yuval Barak-Corren, Victor M. Castro, Franchesca Castro-Ramirez, Tess Lauricella, Nicole Murman, Maria Petukhova, Suzanne A. Bird, Ben Reis, Jordan W. Smoller, Ronald C. Kessler

**Affiliations:** 1 Department of Psychology, Harvard University, Cambridge, Massachusetts; 2 Mental Health Research Program, Franciscan Children’s, Brighton, Massachusetts; 3 Department of Psychiatry, Massachusetts General Hospital, Boston; 4 Department of Psychiatry, Harvard Medical School, Boston, Massachusetts; 5 Department of Biomedical Informatics, Harvard Medical School, Boston, Massachusetts; 6 Department of Bioinformatics, Boston Children’s Hospital, Boston, Massachusetts; 7 Department of Healthcare Policy, Harvard Medical School, Boston, Massachusetts; 8 Center for Precision Psychiatry, Department of Psychiatry, Massachusetts General Hospital, Boston

## Abstract

**Importance:**

Half of the people who die by suicide make a health care visit within 1 month of their death. However, clinicians lack the tools to identify these patients.

**Objective:**

To predict suicide attempts within 1 and 6 months of presentation at an emergency department (ED) for psychiatric problems.

**Design, Setting, and Participants:**

This prognostic study assessed the 1-month and 6-month risk of suicide attempts among 1818 patients presenting to an ED between February 4, 2015, and March 13, 2017, with psychiatric problems. Data analysis was performed from May 1, 2020, to November 19, 2021.

**Main Outcomes and Measures:**

Suicide attempts 1 and 6 months after presentation to the ED were defined by combining data from electronic health records (EHRs) with patient 1-month (n = 1102) and 6-month (n = 1220) follow-up surveys. Ensemble machine learning was used to develop predictive models and a risk score for suicide.

**Results:**

A total of 1818 patients participated in this study (1016 men [55.9%]; median age, 33 years [IQR, 24-46 years]; 266 Hispanic patients [14.6%]; 1221 non-Hispanic White patients [67.2%], 142 non-Hispanic Black patients [7.8%], 64 non-Hispanic Asian patients [3.5%], and 125 non-Hispanic patients of other race and ethnicity [6.9%]). A total of 137 of 1102 patients (12.9%; weighted prevalence) attempted suicide within 1 month, and a total of 268 of 1220 patients (22.0%; weighted prevalence) attempted suicide within 6 months. Clinicians’ assessment alone was little better than chance at predicting suicide attempts, with externally validated area under the receiver operating characteristic curve (AUC) of 0.67 for the 1-month model and 0.60 for the 6-month model. Prediction accuracy was slightly higher for models based on EHR data (1-month model: AUC, 0.71; 6 month model: AUC, 0.65) and was best using patient self-reports (1-month model: AUC, 0.76; 6-month model: AUC, 0.77), especially when patient self-reports were combined with EHR and/or clinician data (1-month model: AUC, 0.77; and 6 month model: AUC, 0.79). A model that used only 20 patient self-report questions and an EHR-based risk score performed similarly well (1-month model: AUC, 0.77; 6 month model: AUC, 0.78). In the best 1-month model, 30.7% (positive predicted value) of the patients classified as having highest risk (top 25% of the sample) made a suicide attempt within 1 month of their ED visit, accounting for 64.8% (sensitivity) of all 1-month attempts. In the best 6-month model, 46.0% (positive predicted value) of the patients classified at highest risk made a suicide attempt within 6 months of their ED visit, accounting for 50.2% (sensitivity) of all 6-month attempts.

**Conclusions and Relevance:**

This prognostic study suggests that the ability to identify patients at high risk of suicide attempt after an ED visit for psychiatric problems improved using a combination of patient self-reports and EHR data.

## Key Points

**Question:**

What is the best method to predict which patients presenting to the emergency department will make a suicide attempt within 1 and 6 months after the visit?

**Findings:**

This prognostic study of 1818 patients found that prediction of suicide attempts in the 1 month and 6 months after a patient visited an emergency department was significantly improved using machine learning models applied to data from a brief patient self-report scale, especially when supplemented with data from patients’ electronic health records and/or clinicians’ assessments.

**Meaning:**

This study suggests that clinicians can improve their ability to identify patients at high risk of suicide by using data from a brief patient self-report scale and electronic health records.

## Teaser

This prognostic study assesses methods of identifying patients at high risk of suicide attempts within 1 and 6 months after patients presented at an emergency department for psychiatric problems using clinician assessments, a brief patient self-report scale, and electronic health records.

## Introduction

Suicide is among the leading causes of death in the US.^1^ Although the mortality rates for most leading causes of death have decreased significantly over the past 100 years, the suicide rate is the same now as a century ago.^2^ Approximately 50% of adults who die by suicide visit a health care professional in the 4 weeks before their death,^3,4^ and approximately 40% visit an emergency department (ED) in the year before their death.^5^ A range of evidence-based interventions exist that can reduce the risk of suicide, but to be cost-effective, many of these interventions require targeting high-risk patients.^6,7^ It is not clear whether clinicians can accurately identify patients at high risk of suicide based on clinical interview alone.^8^

Recent studies suggest that applying machine learning (ML) methods to electronic health records (EHRs) can improve clinicians’ ability to identify patients at hight risk of suicide.^9^ However, critics note that such models have many more false positives than true positives and fail to detect meaningful proportions of the patients who go on to die by suicide.^10^ A separate line of research has suggested that patient self-reports and behavioral data obtained during clinical encounters may help improve clinicians’ ability to identify patients at high risk of suicide attempt.^8,11^ Models that combine information across all these data sources might be the most effective at prediction.^12^

Currently, clinicians use face-to-face interviews to determine patients’ risk of future suicide attempts. Suicide prevention efforts would benefit from an analysis of the comparative strength of predictions based on clinician evaluations, patient self-reports, EHR-based risk scores, and their combinations. Here, we report results of a prospective study designed to do that by predicting suicide attempts within 1 month and 6 months of presentation at an ED for psychiatric problems. This is a high-risk time period in a high-risk segment of the population for whom accurate suicide prediction methods are lacking.^13^

## Methods

### Sample

Participants were patients presenting to the Massachusetts General Hospital ED between February 4, 2015, and March 13, 2017, and seen by the Acute Psychiatry Service because of concerns about psychiatric distress. Inclusion criteria were age 18 years or older and ability to read English. Exclusion criteria were any factors that precluded patient capacity to provide informed consent or complete study procedures (eg, cognitive impairment, florid psychosis, or acute intoxication) as determined by the treating clinician. Study research assistants approached 2532 patients, obtained written informed consent and enrolled 2000 (79.0%), and obtained complete baseline surveys from 1818 unique patients (71.8%). The primary reasons for failure to obtain consent were impairment due to psychiatric symptoms (n = 193); no contact information, precluding follow-up (n = 137); declined (n = 132); language or physical barrier (n = 29); discharged before completing consent (n = 16); family or friend declined (n = 11); and assorted other reasons (n = 14). All study procedures were approved by the Harvard University and Massachusetts General Hospital institutional review boards. Once patients were enrolled, baseline data were collected from 3 sources: (1) evaluations from the treating ED clinician at the end of the clinical encounter on the likelihood that the patient would attempt suicide in the next 1 month and 6 months, (2) a brief (mean [SD], 25 [11] minutes) tablet-based patient self-report questionnaire completed while in the ED, and (3) predicted probabilities of suicide attempt in the next 1 month and 6 months obtained by applying a ML algorithm to patient EHR data available at the time of the ED visit. Follow-up via email and telephone surveys were performed 1 month (n = 1102) and 6 months (n = 1220) later, ending in September 2017. This study followed the Transparent Reporting of a Multivariable Prediction Model for Individual Prognosis or Diagnosis (TRIPOD) reporting guideline.

### Measures

#### Suicide Attempts

The primary dependent variable was the occurrence of a suicide attempt within 1 month and 6 months of the ED visit, as discovered either by patient self-report in follow-up surveys or by review of medical records. Suicide attempts were identified in the medical records via *International Classification of Diseases, Ninth Revision* and *International Statistical Classification of Diseases and Related Health Problems, Tenth Revision* codes using an approach described and validated in prior studies.^14,15^ Suicide attempts were coded as present using an “or” rule in combining EHR data with follow-up survey self-reports. Patients who reported a suicide attempt since the index ED visit in the 1-month follow-up survey were automatically coded as having a 6-month suicide attempt even if they did not complete the 6-month survey.

#### Clinician Evaluations

Prior to hospital discharge, the clinician in the ED responsible for the patient was asked to complete a brief survey that included their rating of the likelihood (0%, 10%, 20%, 30% …100%) that the patient would make a suicide attempt within 1 month and 6 months if untreated and their confidence in that rating, as well as their assessment of the recent (past week) presence of patient suicidal thoughts and attempts (see eAppendix 1 in the Supplement for the full clinician survey). All clinician survey items were used in the clinician prediction model.

#### Patient Self-report Questionnaire

The self-report battery completed in the ED included 67 to 97 (depending on skip logic) questions assessing known risk factors for suicide attempt: sociodemographic characteristics; history of psychiatric symptoms, suicidal thoughts, and suicidal behaviors; family history of psychiatric problems; traumatic life event history; psychological traits; and psychiatric treatment history (eAppendix 2 in the Supplement). The battery also included 2 brief neurocognitive reaction-time tests, the Death Implicit Association Test and the Suicide Stroop Task, both of which have been shown in previous research to predict suicide attempts after ED visits.^8,11^

#### EHR Risk Score

A naive bayesian classifier was applied to each patient’s EHR data (not including the index visit) to generate a separate suicide attempt risk score for 1 month and 6 months after the ED visit. This approach has been used successfully in prior studies to predict suicide attempts after health care visits and is described in detail elsewhere.^14,15^ This score was created separately from the ML approach described below to approximate how such a system could be feasibly implemented. In practice, an EHR-based risk score could be generated for each patient in a health care system, once per month, and combined with patient-generated and clinician-generated information in real time once the patient is in the ED. This is the approach we tested here.

### Statistical Analysis

Statistical analysis was performed from May 1, 2020, to November 19, 2021. We adjusted for the possibility of systematic loss to follow-up by using a 1/p propensity score weight^16^ based on the same stacked generalization ML method that we used to develop the substance model. All baseline variables were included as potential predictors. The substantive analyses were performed with this weighted data set. Missing values were imputed by assigning the median value or most common category. Given that missing data have been found to predict suicide attempts in prior studies,^17,18^ dummy variables for the presence of missing values were created for each section of the self-report battery. Analyses were conducted using SAS, version 9.4 (SAS Institute Inc) and R, version 3.6.3 (R Group for Statistical Computing).

This study was designed to develop and validate a multivariable prediction model that would provide a more accurate method of predicting suicide attempts than what is possible using only clinician evaluations.^19^ Rather than choose a single ML algorithm to develop the models, we used the SuperLearner stacked generalization ML method to combine predictions across a range of algorithms (eAppendix 3 in the Supplement).^20^ The SuperLearner method selects a weighted combination of predicted outcome scores generated via internal cross-validation from a collection (“ensemble”) of user-specified algorithms to generate a single composite predicted outcome score that is guaranteed in expectation to perform at least as well as the best component algorithm according to a prespecified criterion (in our case, maximizing area under the receiver operating characteristic curve [AUC]). The algorithms in the ensemble can be a mix of parametric and flexible ML algorithms, making the SuperLearner method less prone than traditional parametric approaches to model misspecification. The ensemble that we used included logistic regression, penalized regression, splines, decision trees, and gradient boosting (eTable 1 in the Supplement). We used 10-fold cross-validation to avoid overfitting in a 70% training sample to develop the models, and then we evaluated the models in a 30% test sample.

Model performance was evaluated using individual-level predicted probabilities of the outcomes based on each predictor set to generate AUCs in the test sample. The best-performing models were then used to calculate both conditional and cumulative sensitivity and positive predictive value (PPV) across ventiles of the sample distribution along with precision-recall curves.^21^ Predictor importance was evaluated using the Kernel SHAP (Shapley Additive Explanations) method.^22^ A locally estimated scatterplot smoothed (LOESS) calibration curve^23^ (smoothing span = 0.75) was used to visualize model calibration, and we quantified calibration accuracy by calculating the integrated calibration index^24^ based on the LOESS curve as well as the more conventional expected calibration error based on decile binning.^25^

## Results

### Sample Response and Prevalence of Suicide Attempts

Of the 1818 unique participants (1016 men [55.9%]; median age, 33 years [IQR, 24-46 years]; 266 Hispanic patients [14.6%]; 1221 non-Hispanic White patients [67.2%], 142 non-Hispanic Black patients [7.8%], 64 non-Hispanic Asian patients [3.5%], and 125 non-Hispanic patients of other race and ethnicity [6.9%; American Indian or Alaskan Native, Native Hawaiian or Pacific Islander, and other race or ethnicity]) who provided baseline data, 1102 (60.6%) completed the 1-month follow-up survey, and 1220 (67.1%) completed the 6-month follow-up survey. Sociodemographic characteristics of the sample are presented in eTable 2 in the Supplement. The estimated prevalence (SE) of suicide attempts after weighting the data for loss to follow-up was 12.9% (1.0%) as of 1 month (137 of 1102 patients) and 22.0% (1.2%) as of 6 months (268 of 1220 patients) after presenting to the ED.

### Overall Model Performance

Clinician assessment yielded the weakest AUC (SE) in the test sample for both the 1-month and 6-month follow-up periods (1 month, 0.67 [0.04]; 6 months, 0.60 [0.04]) (Table 1). The cross-validated AUC (SE) was higher for EHR data (1 month, 0.71 [0.05]; 6 months, 0.65 [0.04]) and patient self-report (1 month, 0.76 [0.04]; 6 months, 0.77 [0.03]) and highest for models that combined data from patient self-report with other data sources (combined with EHR data: 1 month, 0.77 [0.04]; 6 months, 0.79 [0.03]). Given that use in routine clinical care would require patient self-reports to be kept to a minimum, we explored the implications of restricting the number of survey predictors. A model that combined EHR data with only 20 survey predictors selected by LASSO (Least Absolute Shrinkage and Selection Operator)–penalized logistic regression performed as well as a model with no restriction on the number of survey predictors for 1-month suicide attempts (AUC [SE], 0.77 [0.04] for both models) and nearly as well for 6-month suicide attempts (AUC [SE], 0.78 [0.03] vs 0.79 [0.03]) (eTable 3 in the Supplement).

**Table 1.** Test Sample Receiver Operating Characteristic Curve Area Under the Curves for Predicting 1-Month and 6-Month Suicide Attempt

Source	Area under the curve (SE)	
	At 1 mo	At 6mo
Clinician prediction	0.67 (0.04)	0.60 (0.04)
EHR	0.71 (0.05)	0.65 (0.04)
EHR + clinician prediction	0.75 (0.04)	0.67 (0.04)
Patient self-report	0.76 (0.04)	0.77 (0.03)
Patient self-report + clinician prediction	0.77 (0.04)	0.77 (0.03)
Patient self-report + EHR	0.77 (0.04)	0.79 (0.03)
Patient self-report + EHR + clinician prediction	0.78 (0.04)	0.78 (0.03)

Abbreviation: EHR, electronic health record.

### Precision-Recall Curve

**Figure 1.**
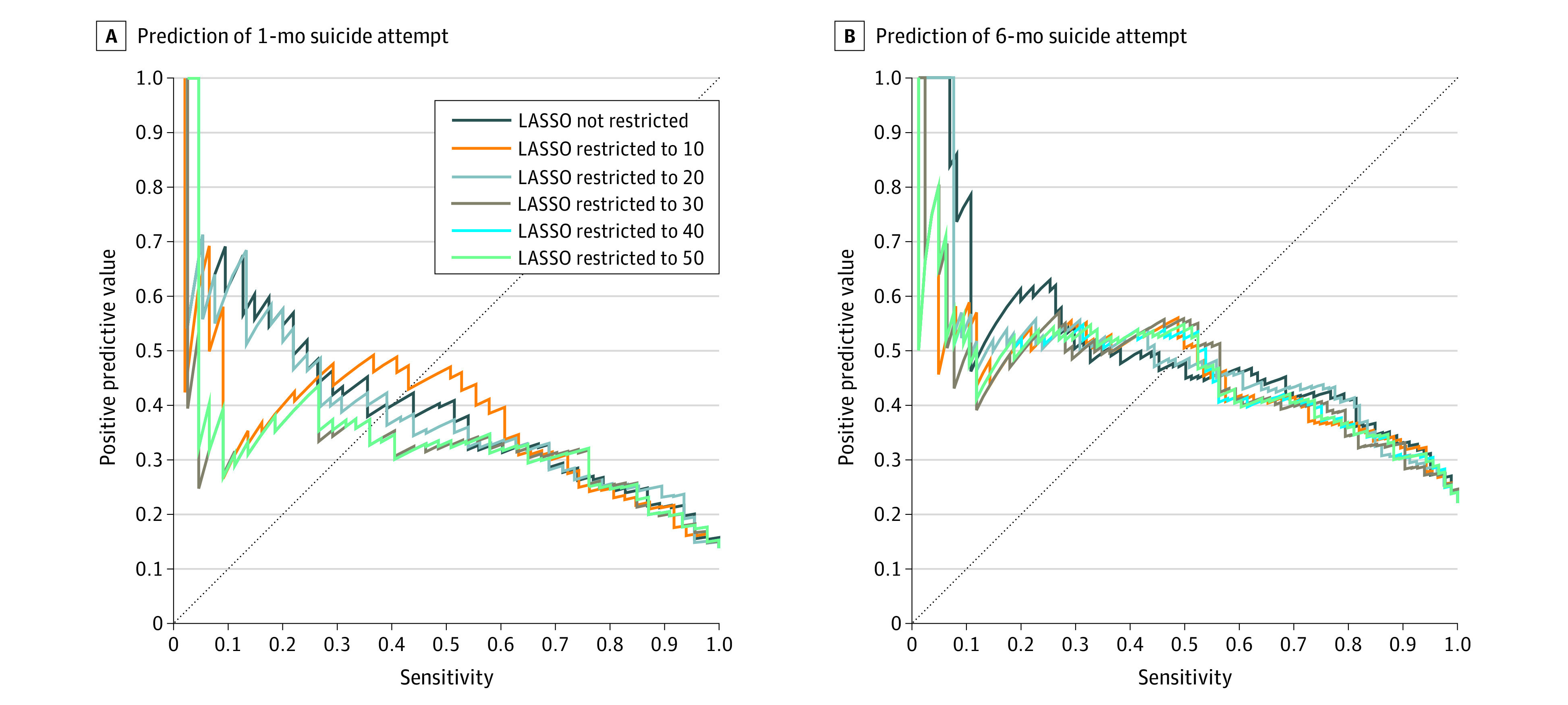
Precision Recall Curves for Predicting Suicide Attempt A, 1-Month suicide attempt. B, 6-Month suicide attempt. LASSO indicates Least Absolute Shrinkage and Selection Operator.

Precision-recall curves were examined to see how PPV varies across levels of sensitivity (Figure 1A and B). We focused on models that combined EHR data with survey predictors and compared model performance depending on the number of survey predictors included in the models. For 1-month suicide attempts, the model restricted to 20 survey predictors and the model with an unrestricted number of survey predictors had a higher PPV (35%-45%) than the other models when sensitivity was in the 0.10 to 0.25 range, whereas the model restricted to 10 survey predictors had the highest PPV (35%-40%) when sensitivity was in the 0.25 to 0.60 range. All models considered were comparable in identifying two-thirds of cases (ie, sensitivity, 0.67) with a PPV of approximately 30% (ie, somewhat more than twice the total-sample prevalence). In predicting 6-month suicide attempts, the model with an unrestricted number of survey predictors had the highest PPV (60%-80%) when sensitivity was in the 0.10 to 0.25 range, and all of the models had a similar performance at higher levels of sensitivity, identifying 55% of cases (ie, sensitivity, 0.55) with a PPV of approximately 45% (ie, about twice the total-sample prevalence).

### Concentration of Risk

We used the model restricted to 20 predictors for 1-month suicide attempts and the model with an unrestricted number of predictors for 6-month suicide attempts. Results from those models were used to create ventiles of predicted suicide attempt risk in the training sample, and then we compared with actual suicide attempt rates based on these predicted probability ranges in the test sample. Positive predictive value and sensitivity were calculated in the test sample both within and cumulatively across these ventiles. For the 1-month data, sensitivity was substantially above the level predicted by chance in the 5 highest-risk ventiles (top 25%). The 28.7% of patients in those ventiles accounted for 64.8% of all 1-month attempts (Table 2). Close to one-third (30.7%) of those high-risk patients made a suicide attempt within 1 month of their ED visit. In contrast, only 6.4% of suicide attempts occurred in the 50% of lower-risk ventiles. Results were broadly similar in the 6-month data, with the 24.1% of patients in the highest 5 risk ventiles accounting for 50.2% of all 6-month attempts and 46.0% of those high-risk patients making a suicide attempt within 6 months of their ED visit (eTable 4 in the Supplement).

**Table 2.** Sensitivity and PPV in the Test Sample of Model Predicting 1-Month Suicide Attempts Based on EHR Plus 20 Patient Self-report Variables

Ventile, %	% of Sample in ventile	1-mo Suicide attempt			
		Within ventile (SE)		Cumulative (SE)	
		Sensitivity	PPV	Sensitivity	PPV
0-5	2.4	5.3 (5.6)	29.8 (17.6)	5.3 (3.7)	29.8 (17.6)
6-10	6.8	21.6 (18.6)	43.1 (11.0)	26.9 (7.0)	39.6 (9.4)
11-15	6.8	12.2 (11.9)	24.4 (9.8)	39.1 (7.6)	33.1 (6.9)
15-20	5.3	12.4 (12.0)	31.9 (11.6)	51.5 (7.7)	32.8 (5.9)
21-25	7.4	13.3 (12.8)	24.4 (8.8)	64.7 (7.3)	30.7 (5.0)
25-30	3.9	4.1 (4.4)	14.3 (9.5)	68.9 (7.1)	28.7 (4.6)
31-35	2.6	2.5 (2.7)	13.0 (12.0)	71.3 (6.9)	27.5 (4.3)
36-40	10.1	9.1 (9.2)	12.3 (5.8)	80.4 (6.0)	24.2 (3.7)
41-45	7.1	9.1 (9.2)	17.6 (8.2)	89.6 (4.5)	23.3 (3.4)
46-50	3.6	4.0 (4.3)	15.4 (10.0)	93.6 (3.6)	22.8 (3.2)
51-55	5.1	0	0	93.6 (3.6)	20.9(3.0)
56-60	4.1	0	0	93.6 (3.6)	19.6 (2.8)
61-65	4.5	1.9 (2.1)	5.8 (5.7)	95.5 (3.1)	18.7 (2.7)
66-70	5.0	0	0	95.5 (3.1)	17.4 (2.5)
71-75	3.6	0	0	95.5 (3.1)	16.6 (2.4)
76-80	6.6	0	0	95.5 (3.1)	15.3 (2.2)
81-85	5.0	2.3 (2.5)	6.2 (6.1)	97.8 (2.2)	14.8 (2.1)
86-90	3.2	2.2 (2.4)	9.2 (8.9)	100.0 (0.0)	14.6 (2.1)
91-95	3.3	0	0	100.0 (0.0)	14.1 (2.0)
96-100	3.3	0	0	100.0 (0.0)	13.6 (2.0)

Abbreviations: EHR, electronic health record; PPV, positive predictive value.

### Calibration and Predictor Importance

The best-performing models had excellent calibration, with an integrated calibration index of 0.043 to 0.051 and an expected calibration error of 0.028 to 0.033 for the 1-month and 6-month models **(**eFigures 1A-B in the Supplement**)**.

**Figure 2.**
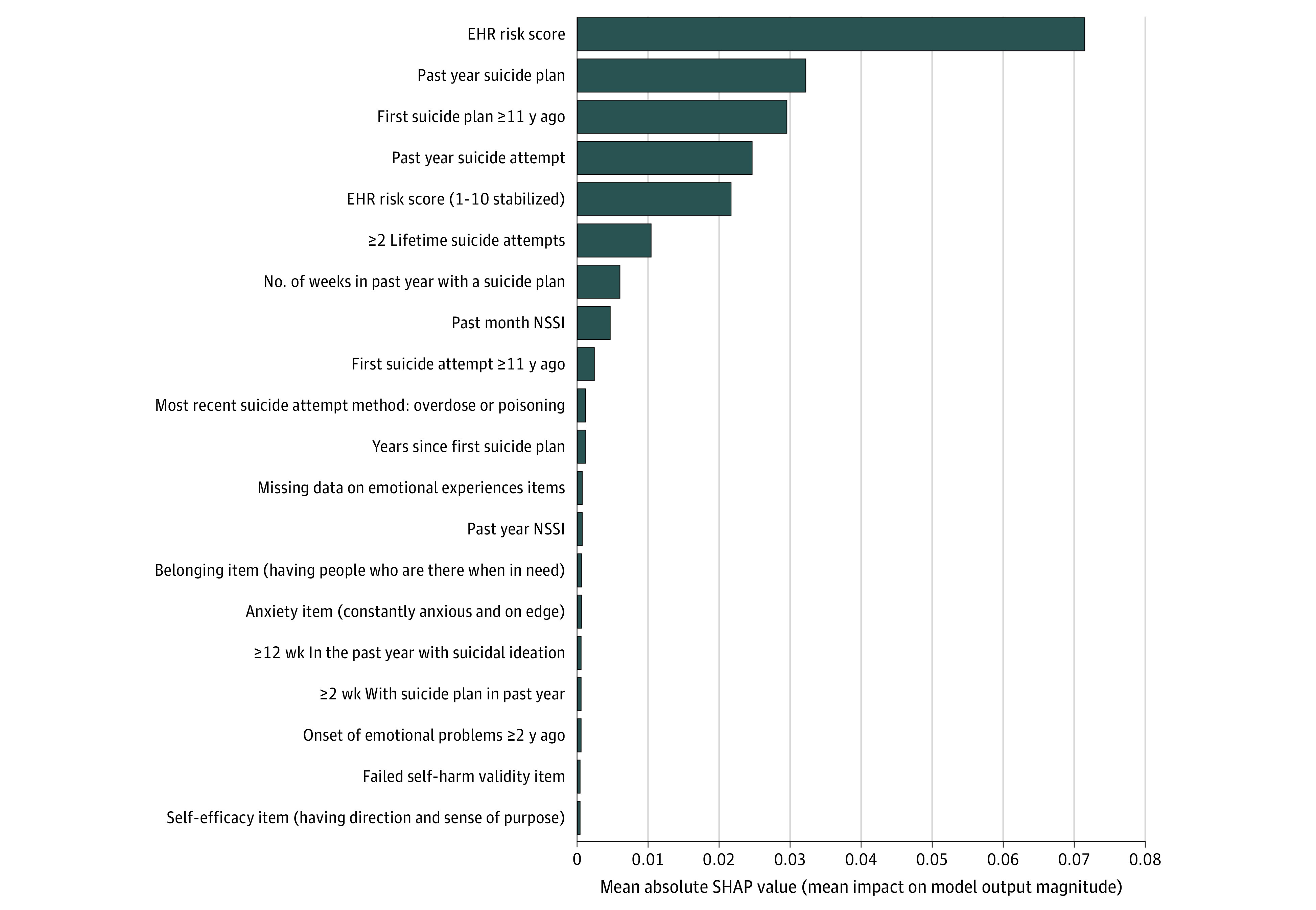
Predictor Variable Importance (Kernel SHAP [Shapley Additive Explanations] Values) in the Test Sample of the Model Predicting 1-Month Suicide Attempts Based on the 20-Predictor Restricted Feature Selection Method EHR indicates electronic health record; NSSI, nonsuicidal self-injury.

The 20 predictors with the highest SHAP values for 1-month suicide attempts were dominated by the EHR score and self-reports about suicidality (Figure 2). This pattern was broadly similar for 6-month prediction (eFigure 2 in the Supplement).

## Discussion

This study has 2 key findings. First, a composite approach that combines patient self-report with data from an EHR-based risk score may improve the prediction of suicide attempts by patients after visiting an ED with levels of accuracy beyond what is possible with clinician assessment or EHR-based risk scores alone. Second, the levels of sensitivity and PPV in the best model are clinically actionable given the known costs and benefits of evidence-based suicide prevention interventions.^6,7^

As noted in the Introduction, half of all people who die by suicide in the US visit a health care setting in the month before their death, providing clinicians an opportunity to identify and intervene with those at risk. In most settings, clinicians make such determinations based on clinical interviews, an approach that is encouraged by clinical practice guidelines.^26^ However, as shown here, clinician assessments are not much better than chance. This result is consistent with other research on the limitations of clinician assessments.^8,27^ However, use of a brief patient self-report battery, especially when combined with other data sources, performed substantially better than clinician assessments alone. The superiority of our models to clinician assessments occurred despite the EHR data used in our model being available for review by clinicians. Presumably, the great complexity of medical histories would make it impossible for a clinician to synthesize EHR data as precisely as can be done in our ML models, even if the severe time constraints imposed on clinicians in busy EDs were not present. In addition, some of the self-report data used in our models contain aspects of patient histories that do not appear in EHRs. Moreover, some patients might not disclose certain details of their histories to clinicians even with probing (eg, childhood maltreatment). Previous research has shown that patients often are more likely to disclose sensitive information of this sort in self-report assessments administered by computer than to clinicians.^28^

Machine learning models developed to predict suicidal outcomes in prior studies have been criticized for having low PPVs (often below 1%) and thus producing too many false positives to justify their use in clinical settings.^10^ Our analyses revealed that, by combining EHR and patients’ self-report data and focusing on an ED sample that was flagged for having psychiatric problems, it is possible to achieve clinically actionable PPVs (above 20%-30%). Our model also performed very well in identifying which patients have a low risk of suicide attempt after their visit—information that can be helpful to consider in clinical decision-making about the need for psychiatric hospitalization for the purposes of patient safety. Even the lowest-risk patients identified by our model had a sufficiently high risk of suicide that low-cost interventions, such as safety planning^29^ or active contact and follow-up,^30^ would be cost-effective^6^; thus, using a risk prediction model to target such interventions would be unnecessary in this population. However, for more intensive interventions, such as psychiatric hospitalization or suicide-focused intensive case management,^31^ using our model to target treatment for the highest-risk patients may substantially improve clinical outcomes and cost-effective use of resources.

### Limitations

This study has several important limitations. First, the study was conducted in only 1 ED, limiting the generalizability of the results. A recent study showed that our EHR-based risk prediction modeling approach has good accuracy across 5 different health care systems throughout the US,^15^ providing encouragement about the external validity of our results, but similar efforts are needed to test the external validity of our model combining EHR data with patient self-report data. Second, a substantial proportion of participants did not complete follow-up surveys. Although we weighted the data to adjust for possible systematic loss to follow-up, the results still may have been biased by such nonresponse. Third, clinicians were asked to make assessments about suicide attempts assuming that the patient went untreated. This scenario was posed to elicit clinicians’ “pure” rating of level of risk without yet knowing what treatment a given patient would receive after visiting the ED. A related problem is that these assessments were likely confounded by decisions about clinical dispositions. For example, clinician assessments of high suicide risk were often accompanied by recommendations for inpatient treatment, which may have decreased the risk of subsequent suicide attempts. However, as available evidence suggests that hospitalization might not have a strong association with prevention of suicides,^32^ this kind of bias might be slight, although nonetheless noteworthy.

## Conclusions

The results of this prognostic study suggest that suicide risk assessments made using EHR-based and self-report–based risk scores may yield relatively accurate and clinically actionable predictions about the risk of suicide attempts by patients after presenting to an ED. These results highlight the need for tests of the implementation of such risk assessment tools to target preventive interventions.

## Notes

Supplement. **eTable 1.** Algorithms Used in the Super Learner Ensemble Machine Learning Analysis **eTable 2.** Sociodemographic Characteristics of the Sample **eTable 3.** Test Sample Receiver Operating Characteristic Curve AUCs for Predicting 1- and 6-Month Suicide Attempts Based on EHR + Patient Self-Report Data With Unrestricted and Restricted Numbers of Survey Predictors **eTable 4.** Sensitivity and PPV in the Test Sample of Model Predicting 6-Month Suicide Attempts Based on EHR + Unrestricted Patient Self-Report Variables **eFigure 1A.** Locally Estimated Scatterplot Smoothed (LOESS) Calibration Curve in the Test Sample of the Model Predicting 1-Month Suicide Attempts Based on EHR + 20 Patient Self-Report Variables **eFigure 1B.** Locally Estimated Scatterplot Smoothed (LOESS) Calibration Curve in the Test Sample of the Model Predicting 6-Month Suicide Attempts Based on EHR + 20 Patient Self-Report Variables **eFigure 2.** Predictor Variable Importance (Kernel SHAP Values) in the Test Sample of the Model Predicting 6-Month Suicide Attempts Based on EHR + 20 Patient Self-Report Variables **eAppendix 1.** Clinician Questionnaire **eAppendix 2.** Baseline Patient Self-Report Survey **eAppendix 3.** SuperLearner Ensemble Methodology
eReferences.
