# Supplementary material for: Prediction of Suicide Attempts Using Clinician Assessment, Patient Self-report, and Electronic Health Records
Source: JAMA Netw Open. 2022 Jan 27;5(1):e2144373. doi: 10.1001/jamanetworkopen.2021.44373 (PMC8796020; doi:10.1001/jamanetworkopen.2021.44373)
Supplement: Supplement. — eTable 1. Algorithms Used in the Super Learner Ensemble Machine Learning Analysis eTable 2. Sociodemographic Characteristics of the Sample eTable 3. Test Sample Receiver Operating Characteristic Curve AUCs for Predicting 1- and 6-Month Suicide Attempts Based on EHR + Patient Self-Report Data With Unrestricted and Restricted Numbers of Survey Predictors eTable 4. Sensitivity and PPV in the Test Sample of Model Predicting 6-Month Suicide Attempts Based on EHR + Unrestricted Patient Self-Report Variables eFigure 1A. Locally Estimated Scatterplot Smoothed (LOESS) Calibration Curve in the Test Sample of the Model Predicting 1-Month Suicide Attempts Based on EHR + 20 Patient Self-Report Variables eFigure 1B. Locally Estimated Scatterplot Smoothed (LOESS) Calibration Curve in the Test Sample of the Model Predicting 6-Month Suicide Attempts Based on EHR + 20 Patient Self-Report Variables eFigure 2. Predictor Variable Importance (Kernel SHAP Values) in the Test Sample of the Model Predicting 6-Month Suicide Attempts Based on EHR + 20 Patient Self-Report Variables eAppendix 1. Clinician Questionnaire eAppendix 2. Baseline Patient Self-Report Survey eAppendix 3. SuperLearner Ensemble Methodology eReferences. [file jamanetwopen-e2144373-s001.pdf]

Supplementary Online Content

Nock MK, Millner AJ, Ross EL, et al. Prediction of suicide attempts using clinician assessment, patient self-report, and electronic health records. *JAMA Netw Open.* 2022;5(1):e2144373. doi:10.1001/jamanetworkopen.2021.44373

eTable 1. Algorithms Used in the Super Learner Ensemble Machine Learning Analysis
eTable 2. Sociodemographic Characteristics of the Sample
eTable 3. Test Sample Receiver Operating Characteristic Curve AUCs for Predicting 1- and 6-Month Suicide Attempts Based on EHR + Patient Self-Report Data With Unrestricted and Restricted Numbers of Survey Predictors
eTable 4. Sensitivity and PPV in the Test Sample of Model Predicting 6-Month Suicide Attempts Based on EHR + Unrestricted Patient Self-Report Variables
eFigure 1A. Locally Estimated Scatterplot Smoothed (LOESS) Calibration Curve in the Test Sample of the Model Predicting 1-Month Suicide Attempts Based on EHR + 20 Patient Self-Report Variables
eFigure 1B. Locally Estimated Scatterplot Smoothed (LOESS) Calibration Curve in the Test Sample of the Model Predicting 6-Month Suicide Attempts Based on EHR + 20 Patient Self-Report Variables
eFigure 2. Predictor Variable Importance (Kernel SHAP Values) in the Test Sample of the Model Predicting 6-Month Suicide Attempts Based on EHR + 20 Patient Self-Report Variables
eAppendix 1. Clinician Questionnaire
eAppendix 2. Baseline Patient Self-Report Survey
eAppendix 3. SuperLearner Ensemble Methodology
eReferences.

This supplementary material has been provided by the authors to give readers additional information about their work.

© 2022 Nock MK et al. *JAMA Network Open.*

**eTable 1.** Algorithms Used in the Super Learner Ensemble Machine Learning Analysis	
**Algorithm**	**Description**
I. Super Learner	Super Learner is an ensemble machine learning approach that uses cross-validation (CV) to select a weighted combination of predicted outcome scores across a collection of candidate algorithms (learners) to yield an optimal combination according to a pre-specified criterion that performs at least as well as the best component algorithm. R package: *Superlearner* (van der Laan, Polley, & Hubbard, 2007).
II. Learners in the Super Learner library	
A. Generalized linear models	Maximum likelihood estimation with flexible link function. R package: *stats* (Nelder & Wedderburn, 1972).
B. Elastic Net	Elastic net is a regularization method that minimizes the problem of overlap among predictors by explicitly penalizing over-fitting with a composite penalty λ{MPP x Plasso + (1- MPP) X Pridge}, where MPP is a mixing parameter penalty with values between 0 and 1 that controls relative weighting between the lasso penalty (Plasso) and the ridge penalty (Pridge). The parameter λ controls the total amount of penalization. The ridge penalty handles multicollinearity by shrinking all coefficients smoothly towards 0 but retains all variables in the model. The lasso penalty allows simultaneous coefficient shrinkage and variable selection, tending to select at most one predictor in each strongly correlated set, but at the expense of giving unstable estimates in the presence of high multicollinearity. The elastic net approach of combining the ridge and lasso penalties has the advantage of yielding more stable and accurate estimates than either ridge or lasso alone while maintaining model parsimony. R package: *glmnet* (Friedman, Hastie, & Tibshirani, 2010).<br>Hyperparameters: α=(0.1, 0.2, 0.3, 0.4, 0.5, 0.6, 0.7, 0.8, 0.9, 1).
C. Splines	
C1. Adaptive splines	Adaptive spline regression flexibly captures both linear and piece-wise non-linear associations as well as interactions among these associations by connecting linear segments (splines) of varying slopes and smooths to create piece-wise curves (basis functions). Final fit is built using a stepwise procedure that selects the optimal combination of basis functions. R package: *earth* (Milborrow, Hastie, Tibshirani, Miller, & Lumley, 2016*)*. Hyperparameters: degree = (1, 3, 5)
C2. Adaptive polynomial splines[a]	Adaptive polynomial splines are like adaptive splines but differ in the order in which basis functions (e.g., linear versus nonlinear) are added to build the final model. R package: *polspline* (Kooperberg, 2015).
D. Decision trees – bagging	Random Forest. Independent variables are partitioned (based on contiguous values) and stacked to build short decision trees that are combined (ensemble) to create an aggregate "forest". Random forest builds numerous trees in bootstrapped samples and generates an aggregate tree by averaging across trees, thereby reducing over-fitting. R package: *ranger* (Wright & Ziegler, 2017). Hyperparameters: max.depth = (6, 8, 8), num.trees = (1550, 1700, 1000), mtry = (12, 4, 20), splitrule = ('gini', 'hellinger', 'extratrees').
E. Decision trees - boosting	
E1. Gradient Boosting Machine	GBMs build a sequential ensemble of shallow successive regression trees that iteratively learn the residuals from prior trees. This is a flexible method, where the number of trees, interaction depth, and shrinkage are leveraged to build flexible models. R package: *Catboost* (Prokhorenkova et al, 2018). Hyperparameters: Iterations=(50, 100), learning_rate=(0.3, 0.8), depth = (8, 10).
E2. Extreme Gradient Boosting	A fast and efficient implementation of gradient boosting. R package: *xgboost* (Chen, & Guestrin, 2016). Hyperparameters: ntrees = (1000, 100, 500, 100, 800), max_depth = (6, 2, 6, 8, 4), shrinkage = (0.001, 0.1, 0.1, 0.1, 0.001), gamma = (0.3, 0.5, 0.0, 0.5, 0.8), minobspernode = (20, 10, 20, 10, 20), colsample_bytree = (0.3, 0.8, 0.5, 0.3, 0.8).
F. Stratified Mean	Arithmetic mean of the outcome computed separately for each of the 9 strata within the sample, where the strata were defined by the nodes of the tree built by the R package *rpart.*
G. Discrete Bayesian Additive Regression Trees Sampler	Fits Bayesian additive regression trees. R package: *dbarts*[73]<br>Hyperparameters: *sigdf = 3, sigquant = 0.90, k = 2.0, power = 2.0, base = 0.95, binaryOffset = 0.0, ntree = 200, ndpost = 1000, nskip = 100.*

© 2022 Nock MK et al. *JAMA Network Open.*

eTable 2. Sociodemographic Characteristics of the Sample

Characteristics	Baseline (*n*=1818) %	1-month (*n*=1102) %	6-month (*n*=1219) %
Age			
18-23	22.0	24.1	24.8
24-29	18.7	21.8	20.3
30-37	20.8	18.3	19.3
38-49	19.8	17.3	18.6
50+	18.8	18.5	16.9
Gender			
Male	56.7	52.2	51.2
Female	41.6	45.8	47.0
Transgender	1.7	2.0	1.8
Race			
Non-Hispanic Black	7.7	7.2	7.0
Non-Hispanic White	67.8	68.9	67.4
Other	24.5	24.0	25.7
Education			
Less than high school diploma, GED, or equivalent	12.4	9.9	11.0
High school diploma, GED, or equivalent	30.4	27.9	27.5
Some post high school education, but no certificate or degree	18.4	17.1	18.4
Post high school technical school certificate or degree (for example, EMT)	5.3	5.3	5.7
2-year college Associate Degree or higher	7.4	8.4	7.0

© 2022 Nock MK et al. *JAMA Network Open.*

**eTable 3.** Test Sample Receiver Operating Characteristic Curve AUCs for Predicting 1- and 6-Month Suicide Attempts Based on EHR + Patient Self-Report Data With Unrestricted and Restricted Numbers of Survey Predictors*				
**Predictors in lasso†**			**AUC (SE)**	
**Min**	**Max**		**1-month**	**6-month**
30	Unrestricted		.77 (.04)	.79 (.03)
5	10		.77 (.04)	.77 (.03)
10	20		.77 (.04)	.78 (.03)
10	30		.76 (.04)	.77 (.03)
10	40		.76 (.04)	.77 (.03)
10	50		.76 (.04)	.77 (.03)

*AUC denotes Area Under the Curve, EHR Electronic Health Record, SE Standard Error.
†Super Learner uses feature selection to minimize over-fitting. In an earlier implementation, both lasso and random forest (ranger) were used as alternative feature selection methods, with the number of features selected in lasso unrestricted and the number in random forests restricted to 30. The AUC in the test sample was higher in the models that used only lasso in predicting both 1-month and 6-month suicide attempts (AUC=.77-.79) than in the model that also used random forests for feature selection (AUC = .76-.78). Based on this result, the final models used only lasso for feature selection.

© 2022 Nock MK et al. *JAMA Network Open.*

eTable 4. Sensitivity and PPV* in the Test Sample of Model Predicting 6-Month Suicide Attempts Based on EHR + Unrestricted Patient Self-Report Variables

		**6-Month Suicide Attempt**			
		**Within Ventile**		**Cumulative**	
**Ventile**	**% of the Sample in Ventile**	**SN† (SE)‡**	**PPV (SE)**	**SN (SE)**	**PPV (SE)**
0-5%	0.5	2.4 (2.5)	100.0 (0.0)	2.4 (1.7)	100.0 (0)
5-10%	6.1	15.0 (13.8)	54.4 (11.4)	17.3 (4.4)	58.0 (10.8)
10-15%	4.1	10.0 (9.8)	54.2 (13.0)	27.4 (5.1)	56.5 (8.3)
15-20%	6.8	10.8 (10.5)	35.3 (10.2)	38.2 (5.6)	48.3 (6.6)
20-25%	6.6	12.0 (11.5)	40.0 (10.4)	50.2 (5.7)	46.0 (5.6)
25-30%	3.1	6.1 (6.3)	44.2 (15.1)	56.3 (5.6)	45.8 (5.2)
30-35%	5.5	8.4 (8.4)	34.0 (10.8)	64.7(5.4)	43.8 (4.7)
35-40%	7.2	12.0 (11.5)	36.7 (9.4)	76.7 (4.8)	42.6 (4.2)
40-45%	5.8	4.7 (4.9)	17.9 (8.2)	81.4 (4.4)	39.4 (3.9)
45-50%	8.9	4.8 (4.9)	11.9 (5.7)	86.2 (3.9)	35.0 (3.5)
50-55%	6.0	5.0 (5.1)	18.4 (8.5)	91.1 (3.2)	33.3 (3.3)
55-60%	7.1	3.9 (4.0)	12.1 (6.6)	95.0 (2.4)	31.1 (3.0)
60-65%	4.2			95.0 (2.4)	29.3 (2.9)
65-70%	7.6	2.6 (2.8)	7.7 (5.2)	97.7 (1.6)	27.2 (2.7)
70-75%	3.2	1.2 (1.2)	8.0 (7.7)	98.8 (1.2)	26.5 (2.6)
75-80%	4.2	.	.	98.8 (1.2)	25.2 (2.5)
80-85%	3.5			98.8 (1.2)	24.2 (2.4)
85-90%	3.2	1.2 (1.3)	6.5 (6.0)	100.0 (0.0)	23.5 (2.4)
90-95%	3.3	.	.	100.0 (0.0)	22.5 (2.3)
95-100%	3.3	.	.	100.0 (0.0)	22.1 (2.2)

*PPV is positive predictive value.
†SN is sensitivity.
‡SE is standard error.

© 2022 Nock MK et al. *JAMA Network Open.*

eFigure 1A. Locally Estimated Scatterplot Smoothed (LOESS) Calibration Curve in the Test Sample of the Model Predicting 1-Month Suicide Attempts Based on EHR + 20 Patient Self-Report Variables

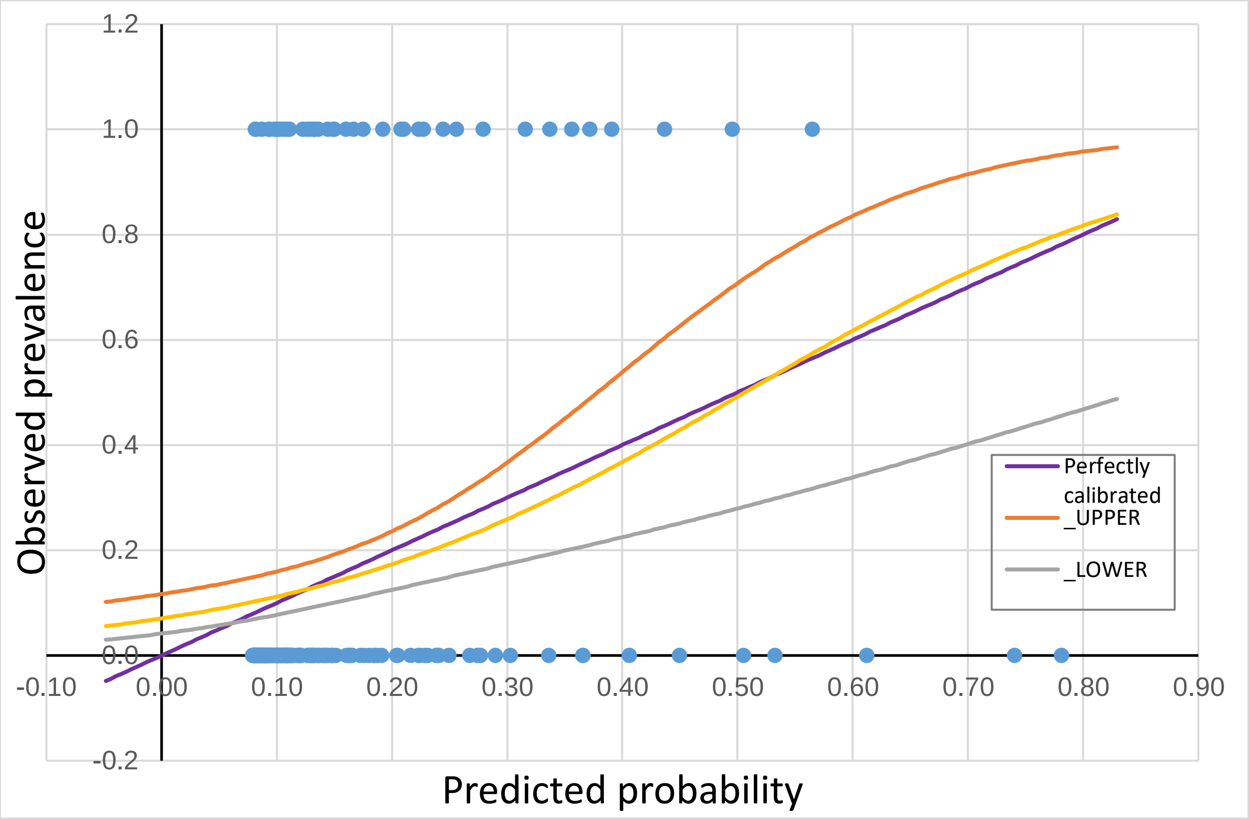

ICI = .043, ECE = .028.

© 2022 Nock MK et al. *JAMA Network Open.*

eFigure 1B. Locally Estimated Scatterplot Smoothed (LOESS) Calibration Curve in the Test Sample of the Model Predicting 6-Month Suicide Attempts Based on EHR + 20 Patient Self-Report Variables

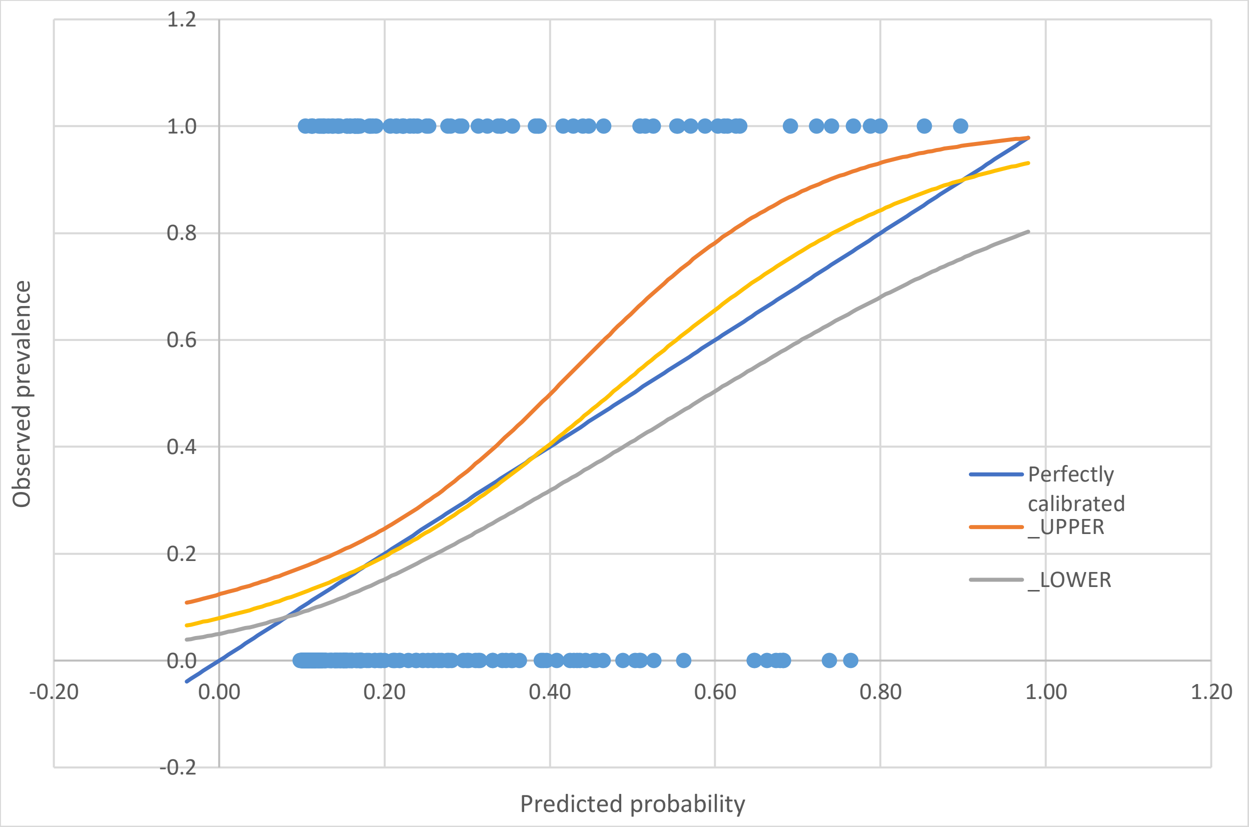

ICI = .05, ECE = .03

© 2022 Nock MK et al. *JAMA Network Open.*

eFigure 2. Predictor Variable Importance (Kernel SHAP Values) in the Test Sample of the Model Predicting 6-Month Suicide Attempts Based on EHR + 20 Patient Self-Report Variables

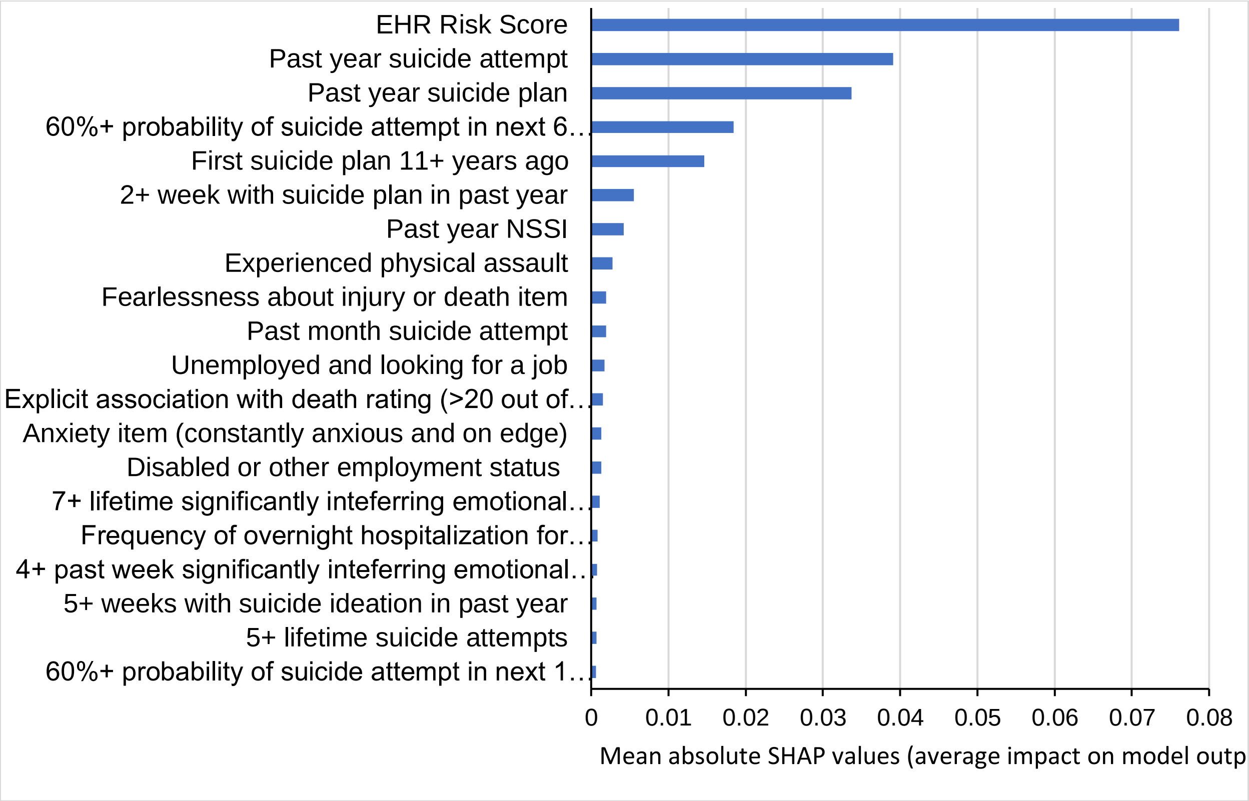

© 2022 Nock MK et al. *JAMA Network Open.*

eAppendix 1. Clinician Questionnaire

(completed by clinician most familiar to patient in APS at time of corresponding interview)

Clinician:

☐ Attending
☐ Resident
☐ Psychiatry Intern
☐ Psychology Intern
☐ Other ____________________

(1) To your knowledge, has this patient experienced **suicide ideation** in the **past week**? ☐ NO ☐ YES

(2) To your knowledge, has this patient made a **suicide attempt** in the **past week**? ☐ NO ☐ YES

(3) Based on your clinical judgment and all you know of this patient, **if untreated**, what is the likelihood that this patient will make a **suicide attempt** in the **next 1 month**?*

0%	10%	20%	30%	40%	50%	60%	70%	80%	90%	100%
○	○	○	○	○	○	○	○	○	○	○

(4) Based on your clinical judgment and all you know of this patient, **if untreated**, what is the likelihood that this patient will make a **suicide attempt** in the **next 6 months**?*

0%	10%	20%	30%	40%	50%	60%	70%	80%	90%	100%
○	○	○	○	○	○	○	○	○	○	○

(5) How confident do you feel about your predictions?

Not at all	A little	Somewhat	Very	Extremely
○	○	○	○	○

(6) Date and Time Completed: ________________ (**Date**) _____:_____ (**Time**) **AM / PM**

***Current research evidence clearly demonstrates that we currently lack the ability to predict future suicide attempts with a high degree of accuracy. Indeed, the purpose of this study is to try to improve on our ability to do so using clinical judgment (which is why we are asking the current questions) and other methods.**

© 2022 Nock MK et al. *JAMA Network Open.*

eAppendix 2. Baseline Patient Self-Report Survey
(Administered on a Tablet in the ED)

Screen 1:

Please enter the Participant ID:

[OPEN ALPHANUMERIC TEXT BOX]

Screen 2:

Thank you for participating in this study. You will be asked to:

- **Complete a brief categorization task**
- **Answer a few questions about yourself**
- **Complete a brief color-naming task**
- **Answer a few more questions about yourself**

Please let the researcher know if you have ANY questions.

Press the "Next" button to begin.

[BUTTON: "Next"]

Screen 3:

[IAT INSTRUCTIONS]

In this task, you will be presented with a set of words to classify into groups. This will take about 5 minutes. Here are the categories and items in this task:

Category	Items
**Me**	I, Myself, Self, Mine
**Not me**	They, Them, Their, Other
**Death**	Suicide, Die, Dead, Deceased
**Life**	Alive, Thrive, Living, Breathing

[BUTTON: "I am ready to begin"]

INSERT **Implicit Association Test** HERE.

© 2022 Nock MK et al. *JAMA Network Open.*

SECTION A: TELL US ABOUT YOURSELF

First, please answer a few questions about yourself.

A1. How old are you?

[NUMERIC KEYPAD] [Programmer: Include constraint >17years old and <100 years old]

A2. What is your gender?

- ○ Male
- ○ Female
- ○ Transgender, Male-to-Female (MTF)
- ○ Transgender, Female-to-Male (FTM)
- ○ Transgender, do not identify as male or female

A3. Are you Spanish/Hispanic/Latino?
(*Check all that apply.*)

○	No
○	Yes, Mexican/Mexican-American/Chicano
○	Yes, Puerto Rican
○	Yes, Cuban
○	Yes, other Spanish/Hispanic/Latino

[Constraint: Restrict selection of both “No” and “Yes” options]

A4. What is your race?
(*Check all that apply.*)

○	White
○	Black or African American
○	American Indiano or Alaskan Native
○	Asian (for example, Chinese, Filipino, Indian)
○	Native Hawaiian or other Pacific Islander
○	Other ____________________________

© 2022 Nock MK et al. *JAMA Network Open.*

A5. What is the highest level of education you completed?

- ○ Less than high school diploma, GED, or equivalent
- ○ High school diploma, GED, or equivalent
- ○ Some post high school education, but no certificate or degree
- ○ Post high school technical school certificate or degree (for example, EMT)
- ○ 2-year college Associate Degree
- ○ 4-year college degree (BA, BS, or equivalent)
- ○ Graduate or professional study

A6. Are you currently married, separated, divorced, widowed, or never married?

- ○ Married
- ○ Separated
- ○ Divorced
- ○ Widowed
- ○ Never married

A7. Do you consider yourself to be:

- ○ Heterosexual/Straight
- ○ Gay/Lesbian/Homosexual
- ○ Bisexual
- ○ Not sure

A8. With whom do you currently live?
(*Check all that apply.*)

- ○ Roommate(s)
- ○ Girlfriend/Boyfriend/Partner
- ○ Spouse
- ○ Children
- ○ Other relatives
- ○ Alone
- ○ Homeless/Shelter
- ○ Other:________________________

© 2022 Nock MK et al. *JAMA Network Open.*

A9. Which option below best describes your current work situation?

- ○ Working now
- ○ Temporarily laid off, sick leave or maternity leave
- ○ Looking for work, unemployed
- ○ Retired
- ○ Disabled, permanently or temporarily
- ○ Keeping house
- ○ Student
- ○ Other:______________________________

CKPT: If A9: "temporarily laid off"/"looking for work"/ "retired"/"disabled" / "other" → GO TO A9a.
All others go to A10.

A9a. How long has it been since you worked?

- ○ Within past 6 months
- ○ 7 months to 11 months
- ○ 1 year to 5 years
- ○ More than 5 years

A10. What is your best estimate of your [if married: **"and your spouse's"] total income from all sources, before taxes, in 2014?**

- ○ $0 - 20,000
- ○ $21,000 - $40,000
- ○ $41,000 - $60,000
- ○ $61,000 - $80,000
- ○ $81,000 - $100,000
- ○ >$100,000

A11. Please answer "C" for this question.

- ○ A
- ○ B
- ○ C
- ○ D
- ○ E
- ○ F

© 2022 Nock MK et al. *JAMA Network Open.*

SECTION B: Emotional Problems

The next questions are about emotional problems you may have had.

B1. Have you had problems with the following issues that lasted more than 1 month and were so bad that they caused problems for you at work/school, with your family, or friends? If so, when was the last time?

	Past week	Past year (not past week)	More than a year ago	Never
a) you were so sad or depressed that you couldn't concentrate, had trouble eating or sleeping, or didn't want to do things you usually enjoy (FILL: having depression)	○	○	○	○
b) you were hyper or manic; that is, much more excited and full of energy than usual, your mind went too fast, and you did things that were unusual for you (for example, taking risks, spending too much money) (FILL: being hyper or manic)	○	○	○	○
c) you had insomnia, or problems falling asleep, staying asleep, or waking up too early (FILL: having insomnia)	○	○	○	○
d) you had repeated attacks of fear or panic when all of a sudden you felt very frightened, anxious, or uneasy (FILL: having attacks of fear or panic)	○	○	○	○
e) you had repeated attacks of anger when all of a sudden you lost control and broke or smashed something, hit or tried to hurt someone, or threatened someone (FILL: having attacks of anger)	○	○	○	○
f) Please answer "Never" for this question.	○	○	○	○
g) after a traumatic experience you had frequent upsetting memories or dreams, felt jumpy, distant, or depressed, and had trouble sleeping or concentrating. (*Do not report the most recent time a traumatic experience occurred, but rather the most recent time you had reactions of the sort described here to a traumatic experience.*) (FILL: having trouble after a traumatic experience)	○	○	○	○

© 2022 Nock MK et al. *JAMA Network Open.*

B1. Have you had problems with the following issues that lasted more than 1 month and were so bad that they caused problems for you at work/school, with your family, or friends? If so, when was the last time?

	Past week	Past year (not past week)	More than a year ago	Never
h) you were so agitated, anxious, or worried that you couldn't relax, sleep, or concentrate, and you couldn't function normally (FILL: having anxiety)	○	○	○	○
i) you used alcohol so much that it got you in trouble at work/school or with family/friends, or your use was out of control (FILL: trouble with alcohol)	○	○	○	○
j) you used drugs (for example, marijuana, cocaine, etc.) so much that it got you in trouble at work/school or with family/friends, or your use was out of control (FILL: trouble with drugs)	○	○	○	○
k) you had unusual experiences like seeing or hearing things that other people couldn't see or hear. (*Do not include times when you were half-asleep or drinking alcohol or taking drugs.*) (FILL: seeing or hearing strange things)	○	○	○	○
l) you had strange thoughts like believing your mind was being controlled by outside forces, that someone or something was sending you special signs (like through the TV), or that someone or something was plotting to harm you (FILL: having strange thoughts)	○	○	○	○

CKPT:

1. **AT LEAST 1 IN B SERIES REPORTED IN LIFETIME → GO TO B2**
2. **ALL OTHERS GO TO SECTION C**

B2. You reported (IF 1: FILL)/ (IF 2: FILL and FILL)/ (IF 3: FILL and FILL and FILL/ IF 4+: some of the above problems, like FILL, FILL and FILL). About how old were you when (IF 1: this problem/ IF 2+: any of these problems) first started?

____ Years old [NUMERIC KEYPAD] [Constraint: ≤ current age provided in A1]

CKPT.B2

1. AT LEAST 1 IN B SERIES REPORTED IN PAST YEAR → GO TO B3
2. ALL OTHERS GO TO SECTION C

© 2022 Nock MK et al. *JAMA Network Open.*

B3. About how many months out of 12 in the past year did you have (this problem/ any of these problems)?
(*Your best estimate is fine.*)

_____ Number of months [NUMERIC KEYPAD] [Constraint: ≤ 12]

© 2022 Nock MK et al. *JAMA Network Open.*

SECTION C: SELF-HARM

The next questions are about thoughts of hurting yourself.

C1. Did you <u>ever in your life</u> have thoughts of killing yourself?

○ Yes
○ No ⟶ GO TO C2

C1a. About how old were you the very first time you had thoughts of killing yourself?

_______ **Years old** [NUMERIC KEYPAD] [Constraint: ≤ current age provided in A1]

C1b. About how many <u>weeks in the past year</u> did you think of killing yourself?
(*You can use any number between 0 and 52. Your best estimate is fine.)*

__________ **Number of weeks** [NUMERIC KEYPAD] [Constraint: ≤ 52]

CKPT: If C1b=0→ GO TO C3.
If C1b>0→ GO TO C1c.

C1c. When was the <u>last time</u> you had thoughts of killing yourself?

○ Past week
○ 2-4 weeks ago
○ 1-3 months ago
○ 4-6 months ago
○ 7-12 months ago

CKPT.C1. GO TO C3

C2. Did you ever wish you were dead or would go to sleep and never wake up?
○ Yes
○ No ⟶ GO TO C5

C2a. About how old were you the very first time you wished you were dead or would go to sleep and never wake up?

_______ **Years old** [NUMERIC KEYPAD] [Constraint: ≤ current age provided in A1]

C2b. About how many <u>weeks in the past year</u> did you wish you were dead or would go to sleep and never wake up?
(*You can use any number between 0 and 52. Your best estimate is fine.*)

__________ **Number of weeks** [NUMERIC KEYPAD] [Constraint: ≤ 52]

CKPT: If C2b=0→GO TO C3
If C2b>0→GO TO C2c

© 2022 Nock MK et al. *JAMA Network Open.*

C2c. When was the <u>last time</u> you wished you were dead or would go to sleep and never wake up?

- ○ Past week
- ○ 2-4 weeks ago
- ○ 1-3 months ago
- ○ 4-6 months ago
- ○ 7-12 months ago

C3. Did you ever make a plan to kill yourself?

- ○ Yes
- ○ No ⟶ GO TO C4

C3a. About how old were you the very first time you made a plan to kill yourself?

______ **Years old** [NUMERIC KEYPAD] [Constraint: ≤ current age provided in A1]

C3b. About how many <u>weeks in the past year</u> did you have a plan to kill yourself?
(*You can use any number between 0 and 52. Your best estimate is fine.)*

__________ **Number of weeks** [NUMERIC KEYPAD] [Constraint: ≤ 52]

CKPT: If C3b=0→ GO TO C4.
If C3b>0→ GO TO C3c.

C3c. When was the <u>last time</u> you had a plan to kill yourself?

- ○ Past week
- ○ 2-4 weeks ago
- ○ 1-3 months ago
- ○ 4-6 months ago
- ○ 7-12 months ago

C4. Did you ever make a suicide attempt (that is, purposefully hurt yourself with at least some intent to die)?

- ○ Yes
- ○ No ⟶ GO TO C5

C4a. About how old were you the very first time you made a suicide attempt?

______ **Years old** [NUMERIC KEYPAD] [Constraint: ≤ current age provided in A1]

C4b. How many different suicide attempts did you ever make?

[NUMERIC KEYPAD] [Constraint: ≥ 1]

© 2022 Nock MK et al. *JAMA Network Open.*

CKPT: IF C4a=A1 OR (A1 – 1) → GO TO C4c.
IF C4a<A1-1 and C4b>1 → GO TO C4c
IF C4a<A1-1 and C4b=1 → GO TO C4d
ALL OTHERS GO TO C5

C4c. When was the <u>last time</u> you made a suicide attempt?

- ○ Past week
- ○ 2-4 weeks ago
- ○ 1-3 months ago
- ○ 4-6 months ago
- ○ 7-12 months ago
- ○ More than 1 year ago

C4d. Which method(s) did you use for your suicide (if C4b=1: **"attempt"/** if C4b>1: **"attempts")?**
(Check all that apply.)

- ○ Overdose of medications
- ○ Overdose of illegal drugs
- ○ Poisoning with a household substance or gas
- ○ Hanging
- ○ Suffocation (for example, plastic bag over head)
- ○ Drowning
- ○ Cutting or stabbing
- ○ Gunshot
- ○ Jumping from a high place
- ○ Motor vehicle crash
- ○ Any other method

CKPT:
IF C4c="past week" or "2-4 weeks ago" and C4b=1, GO TO CKPT C4d0
IF C4c="past week" or "2-4 weeks ago" and C4b>1, GO TO C4d0
All others GO TO C5

C4d0. Which method(s) did you use for your <u>most recent</u> suicide attempt? (Check all that apply)

- ○ Overdose of medications
- ○ Overdose of illegal drugs
- ○ Poisoning with a household substance or gas
- ○ Hanging
- ○ Suffocation (for example, plastic bag over head)
- ○ Drowning
- ○ Cutting or stabbing
- ○ Gunshot
- ○ Jumping from a high place
- ○ Motor vehicle crash
- ○ Any other method

CKPT: If >1 response selected in C4d0, GO TO C4d0i. All others GO TO CKPT C4d0

© 2022 Nock MK et al. *JAMA Network Open.*

CKPT C4d0:
IF C4d0="Overdose of medications" or "Overdose of illegal drugs" or "Poisoning with a household substance or gas" → GO TO C4d1
IF C4d0="Hanging" → GO TO C4d2
IF C4d0="Suffocation (for example, plastic bag over head)" or "Drowning" → GO TO C4d3
IF C4d0="Cutting or stabbing" → GO TO C4d4
IF C4d0="Gunshot" → GO TO C4d5
IF C4d0="Jumping from a high place" or "Motor vehicle crash" → GO TO C4d6
IF C4d0="Any other method" → GO TO C4d7

C4d0i. Which method from your most recent suicide attempt caused the most severe injuries to you?
[NOTE TO PROGRAMMER: IDEALLY, WE WOULD LIKE TO PIPE IN ONLY THE RESPONSES SELECTED IN THE PRIOR QUESTION. IF THIS IS NOT POSSIBLE, SHOW THE FULL LIST]

- ○ Overdose of medications
- ○ Overdose of illegal drugs
- ○ Poisoning with a household substance or gas
- ○ Hanging
- ○ Suffocation (for example, plastic bag over head)
- ○ Drowning
- ○ Cutting or stabbing
- ○ Gunshot
- ○ Jumping from a high place
- ○ Motor vehicle crash
- ○ Any other method

CKPT C4d0i:
IF C4d0i="Overdose of medications" or "Overdose of illegal drugs" or "Poisoning with a household substance or gas" → GO TO C4d1
IF C4d0i="Hanging" → GO TO C4d2
IF C4d0i="Suffocation (for example, plastic bag over head)" or "Drowning" → GO TO C4d3
IF C4d0i="Cutting or stabbing" → GO TO C4d4
IF C4d0i="Gunshot" → GO TO C4d5
IF C4d0i="Jumping from a high place" or "Motor vehicle crash" → GO TO C4d6
IF C4d0i="Any other method" → GO TO C4d7

C4d1. What are the most severe kind of injuries you had as a result of that suicide attempt?
(Please check one)

- ○ Fully awake and alert
- ○ Slowed-speech and movement but responsive to questions/minimal medical problems or treatment
- ○ Severely reduced awareness, or some injury (e.g. mouth burns)
- ○ Hospitalization - vital signs severely affected or passed out
- ○ Passed out- major medical problems such as kidney failure, needed blood transfusion
- ○ Unknown

C4d2. What are the most severe kind of injuries you had as a result of that suicide attempt?
(Please check one.)

© 2022 Nock MK et al. *JAMA Network Open.*

- ○ No damage
- ○ Simple rope burns
- ○ More serious injuries with same-day medical treatment
- ○ Lost consciousness, stopped breathing and resuscitation required
- ○ Paralysis or other spinal cord injury
- ○ Unknown

C4d3. What are the most severe kind of injuries you had as a result of that suicide attempt? *(Please check one.)*

- ○ No damage
- ○ Awake-some breathing problems but no resuscitation needed
- ○ Awake-minimal to moderate efforts at resuscitation needed
- ○ Awake-serious effort at resuscitation needed
- ○ Unconscious-massive efforts at resuscitation necessary for revival
- ○ Unknown

C4d4. What are the most severe kind of injuries you had as a result of that suicide attempt? *(Please check one.)*

- ○ Surface scratches; none or minor bleeding; little or no wound care required
- ○ Moderate bleeding; simple wound care required
- ○ Bleeding of major vessel, danger of serious blood loss without suturing; <u>same-day medical care</u>
- ○ Extensive blood loss; suturing, blood replacement and tendon repair; <u>hospitalization required</u>
- ○ Extensive blood loss; went into shock
- ○ Unknown

C4d5. What are the most severe kind of injuries you had as a result of that suicide attempt? *(Please check one.)*

- ○ No damage
- ○ Flesh wounds with powder burns
- ○ Bullet lodged in arm or leg - minor bleeding
- ○ Bullet lodged in stomach or chest - major bleeding
- ○ Bullet to head area
- ○ Unknown

C4d6. What are the most severe kind of injuries you had as a result of that suicide attempt? *(Please check one.)*

- ○ Minor bruises only - no treatment necessary
- ○ Sprains or minor injuries - no bone, or tendon damage; no internal bleeding, or brain damage

© 2022 Nock MK et al. *JAMA Network Open.*

○ Fractured arms or legs- needed a cast but recovered completely
○ Major bone and/or tendon damage in multiple areas and internal bleeding
○ Major damage in skull neck or spinal cord paralysis expected
○ Unknown

C4d7. What are the most severe kind of injuries you had as a result of that suicide attempt. *(Please check one.)*

______________________ **[**text**]**

C5. Did you ever do something to hurt yourself on purpose, but <u>without</u> wanting to die (for example, cutting yourself, hitting yourself, or burning yourself)?

○ Yes
○ No ⟶ GO TO C6v

C5a. About how old were you the very first time you did something to hurt yourself on purpose, but <u>without</u> wanting to die?

______ **Years old** [NUMERIC KEYPAD] [Constraint: ≤ current age provided in A1]

C5b. About how many times in your life did you do something to hurt yourself on purpose, but <u>without</u> wanting to die?

○ 1-2 times
○ 3-5
○ 6-10
○ 11-20
○ 21-30
○ 31-50
○ 51-100
○ 101 or more times

C5c. When was the <u>last time</u> you did something to hurt yourself on purpose, but <u>without</u> wanting to die?

○ Past week
○ 2-4 weeks ago
○ 1-3 months ago
○ 4-6 months ago
○ 7-12 months ago
○ More than 1 year ago

C6v. Did you ever make a suicide attempt (that is, purposefully hurt yourself with at least some intent to die)?

○ Yes
○ No ⟶ GO TO C6

© 2022 Nock MK et al. *JAMA Network Open.*

C6cv. When was the <u>last time</u> you made a suicide attempt?

- ○ Past week
- ○ 2-4 weeks ago
- ○ 1-3 months ago
- ○ 4-6 months ago
- ○ 7-12 months ago
- ○ More than 1 year ago

C6. Please answer "C" for this answer.

- ○ A
- ○ B
- ○ C ⟶ GO TO Stroop
- ○ D
- ○ E
- ○ F

<u>INSERT **Stroop Test** HERE.</u>

© 2022 Nock MK et al. *JAMA Network Open.*

SECTION D: FAMILY HISTORY

D1. The next questions are about the mental health of your biological parents (not stepparents). We want to know about problems that ever occurred, so answer even for people who are no longer alive.

Did either of your biological parents ever…

	Yes	No	Don't know
a. have times lasting 2 weeks or longer when they were so depressed, they couldn't concentrate, felt worthless, or felt their life was not worth living?	○	○	○
b. Please answer "Don't Know" for this question.	○	○	○
c. have manic episodes lasting several days or longer when they were excited, full of energy, and did dangerous or embarrassing things? *(Do not include times due to using drugs or alcohol.)*	○	○	○
d. have anxiety attacks when they suddenly felt terrified for no good reason and would either shake, sweat, or have other physical symptoms?	○	○	○
e. have anger attacks when they suddenly lost control and "blew up" for no good reason, either yelling, breaking things, or hurting people?	○	○	○
f. have long periods of time where they were more agitated, anxious, or worried than other people so that they couldn't relax, couldn't concentrate, or couldn't function normally?	○	○	○
g. have significant problems with alcohol or drugs that caused problems at home, work, or with friends, or that was out of control?	○	○	○
h. attempt suicide or die by suicide?	○	○	○
i. go to prison?	○	○	○

© 2022 Nock MK et al. *JAMA Network Open.*

SECTION E: YOUR LIFE HISTORY

The next questions are about your childhood.

E1. How often did you have each of the following experiences up through age 18?

	Very often	Often	Sometimes	Rarely	Never
a. An adult in your house swore at you, insulted you, or acted in a way that made you afraid you would be physically hurt	○	○	○	○	○
b. An adult in your house pushed, grabbed, or slapped you, or hit you so hard that it left a mark	○	○	○	○	○
c. Your parents or the people who raised you yelled at or insulted each other	○	○	○	○	○
d. Your parents or the people who raised you pushed, grabbed, or slapped each other, or hit each other so hard that it left a mark	○	○	○	○	○
e. People in your family did not look out for you or support you	○	○	○	○	○
f. People in your family didn't worry about whether you had adequate food or clothing or medical care	○	○	○	○	○
g. Someone older than you touched or fondled you in a sexual way	○	○	○	○	○

© 2022 Nock MK et al. *JAMA Network Open.*

E2. The next questions are about highly stressful experiences that might have happened to you at <u>any time in your life</u>.

How many times did you experience each of the following?

	0	1	2-4	5-9	10 or more
a. Serious physical assault (for example, beaten up or mugged)	○	○	○	○	○
b. Sexual assault or rape	○	○	○	○	○
c. You witnessed someone else being badly beaten or killed	○	○	○	○	○

© 2022 Nock MK et al. *JAMA Network Open.*

E3. The next questions are about you.

How well does each of the following statements describe you?

	Exactl y like me	A lot like me	Somew hat like me	A little like me	Not at all like me
a. When I experience emotions, I feel them very intensely	○	○	○	○	○
b. I am hopeless about the future	○	○	○	○	○
c. I feel like time is running out for me	○	○	○	○	○
d. I feel trapped by my problems	○	○	○	○	○
e. I am constantly anxious and on edge	○	○	○	○	○
f. Life is impossible if things don't change right away	○	○	○	○	○
g. When I am upset, I often act without thinking	○	○	○	○	○
h. I am a burden to others	○	○	○	○	○
i. I feel like I don't belong	○	○	○	○	○
j. I am not afraid of injury or death	○	○	○	○	○
k. I have people who care about me and are there for me whenever I need them	○	○	○	○	○
l. I have a sense of direction and purpose in life	○	○	○	○	○
v. Please answer "Not at all like me" for this question.	○	○	○	○	○

© 2022 Nock MK et al. *JAMA Network Open.*

SECTION F: TREATMENT

The next questions are about treatment for emotional or behavioral problems.

F1. During the past 12 months (52 weeks), how many times…

**a.** did you have a talk therapy session with a psychologist, psychiatrist, or other counselor for emotional or behavioral problems?	[Numeric keypad]
**b.** did you fill a prescription for antidepressant medication (that is, how many times did you go to the pharmacy to pick up this medication)?	[Numeric keypad]
**c.** did you come to an emergency room for help with emotional or behavioral problems (not including this visit)?	[Numeric keypad]
v. please answer "0" for this question.	[Numeric keypad]
**d.** were you admitted for an overnight stay at a hospital for the treatment of emotional or behavioral problems (indicate the number of separate times this happened, not including this visit)?	[Numeric keypad]

CKPT.F1
If F1d>0, go to F1e
If F1d=0, go to the next section

F1e. During the past 12 months, how many total nights did you stay in a hospital for the treatment of emotional or behavioral problems (not including this visit)?

[NUMERIC KEYPAD]

© 2022 Nock MK et al. *JAMA Network Open.*

SECTION G: Final Questions

G1. To what extent do you think of <u>death</u> as being "like you" versus "not like you?"

(Slide ruler with "not like me" on left end and "like me" on right end)

G2. What are the chances that you will make a suicide attempt in the <u>next 1 month</u>?

- ○ 0%
- ○ 10%
- ○ 20%
- ○ 30%
- ○ 40%
- ○ 50%
- ○ 60%
- ○ 70%
- ○ 80%
- ○ 90%
- ○ 100%

G3. Please answer "50%" for this question.

- ○ 0%
- ○ 10%
- ○ 20%
- ○ 30%
- ○ 40%
- ○ 50%
- ○ 60%
- ○ 70%
- ○ 80%
- ○ 90%
- ○ 100%

G4. What are the chances that you will make a suicide attempt in the <u>next 6 months</u>?

- ○ 0%
- ○ 10%
- ○ 20%
- ○ 30%

© 2022 Nock MK et al. *JAMA Network Open.*

- ◯ 40%
- ◯ 50%
- ◯ 60%
- ◯ 70%
- ◯ 80%
- ◯ 90%
- ◯ 100%

© 2022 Nock MK et al. *JAMA Network Open.*

eAppendix 3. SuperLearner Ensemble Methodology

The SuperLearner ensemble methodology, also known as stacking (Wolpert 1992), is a form of supervised learning in which multiple ways of predicting an outcome variable are evaluated and combined (van der Laan, Polley, and Hubbard 2007; Polley, Rose, and van der Laan 2011). Each way of predicting an outcome variable is known as an *estimator* or *learner*, and consists of up to four components:

1. Estimation algorithm: a prediction method that estimates ("learns") a mapping f(•) from the predictor variables (X) to the outcome variable Y.
2. Hyperparameter configuration: the set of tuning settings for an estimation algorithm that must be pre-specified rather than learned from the data.
3. Feature selection: optional identification of a subset of predictors that will be provided to the estimation algorithm, or simply all available features.
4. Feature transformations: optionally any transformations of the original predictor space, such as dimensionality reduction, the addition of interaction terms, imputation of missing values, or the calculation of basic functions.

One estimator might be logistic regression with no further customization. Another estimator might be random forest configured to estimate 1,000 trees (a hyperparameter), provided with predictors that have a Pearson correlation coefficient p-value of 0.2 or less (feature selection). Another estimator might be ordinary least squares (OLS) provided with all predictors. All two-way interactions and squared terms have been added to the predictor list (feature transformations).

Estimators are evaluated through cross-validation, which entails partitioning the analyzed dataset into distinct subsets known as folds. All folds except one are combined into a training set, and each estimator is provided with the training set to estimate the mapping f(•) from the predictors (X) to the outcome (Y). The estimator's learned function is then applied to the remaining fold, known as the test set, and evaluated for its accuracy using a pre-specified loss function such as mean-squared error, negative log likelihood loss, or 1 - AUC. Evaluating performance on a held-out test set, which was not used to estimate the estimator's parameters, is important for identifying any overfitting. Each fold typically serves as the test set once, and the performance estimates are averaged to determine the cross-validated loss for each estimator.

In the simplest case, the estimator with the lowest cross-validated loss is chosen. This is known as the cross-validation selector or discrete SuperLearner, and has been proven to perform asymptotically as well as a selection strategy based on understanding the true data distribution (oracle inequality) (Van Der Laan and Dudoit 2003). The implication is that there is little danger in using cross-validation to choose the best-performing estimator among a set of varied prediction strategies. Rather than only trying our personal favorite method or borrowing a recommendation from the literature, we can empirically validate multiple methods and allow the cross-validation procedure to report which method has been most successful in minimizing our loss function on the dataset at hand.

Choosing a single estimator may leave valuable performance on the table. Instead, it may be advantageous to combine the predictions of multiple estimators, possibly improving on the bias-variance tradeoff of any single estimator. Super Learning, or stacked ensembling, leverages the

© 2022 Nock MK et al. *JAMA Network Open.*

cross-validation approach described above to identify an optimal combination of individual estimators that minimizes the chosen loss function (e.g., mean-squared error). It does so by taking the test set data, established when each cross-validation fold is used for evaluation of the estimators, and "stacking" (appending) those test sets into a combined dataset with the same number of observations as the original data. In this stacked dataset, the predicted value of each algorithm becomes a predictor (column), a form of coordinate transformation from the original predictor space, and we call this the "Z" matrix. A metalearner algorithm is then applied to the Z matrix, which learns a function g(•) that maps the test set predictions of each estimator to the outcome variable (Y).

The most common metalearner algorithm is a convex combination of the columns of Z. In that case, it is a simple convex optimization problem to identify the set of non-negative weights that can be applied to the Z matrix to minimize the chosen loss function for predicting Y. This approach is often implemented by using non-negative least squares to estimate non-negative but otherwise unbounded weights and then rescaling those weights to sum to 1. Convex weights are beneficial for a number of reasons, including that their minimal data-adaptivity reduces the risk of overfitting, they ensure that the ensemble prediction falls within the convex hull of the original estimators' predictions, and they induce sparsity (i.e., 1 or more predictors may have a weight of 0 in the ensemble, simplifying the prediction).

More complex metalearners might be used as an alternative to the convex combination, such as a random forest or highly adaptive lasso (Benkeser and van der Laan 2016). They risk overfitting to the Z matrix, but if their complexity can be appropriately controlled their incorporation of interaction terms holds the promise of identifying regions of the estimator space (Z) where certain estimators are more accurate than others. As a result, they may be able to achieve even higher predictive performance (LeDell and Poirier 2020).

Once the metalearner estimator has been trained, each constituent estimator is optionally retrained on the full dataset as the final step. This gives each estimator a slight performance boost by not taking out a rotated test set, as was done during the earlier cross-validation. Alternatively, it is possible to skip this final retraining of the constituent estimators; and instead, use each version of the estimators trained on the separate training sets. For example, with 10-fold cross-validation, there would be a copy of each estimator trained on the 10 versions of a training set. The prediction of each copy of a given estimator would be averaged before going into the metalearner to yield the ensemble prediction.

In this work, we use the SuperLearner R package (Polley et al. 2019), although an alternative implementation sl3 is under development (Coyle et al. 2021). Additional details on the SuperLearner algorithm and best practices are available (Naimi and Balzer 2018; Kennedy 2017; Polley and van der Laan 2010).

© 2022 Nock MK et al. *JAMA Network Open.*

eReferences.

Benkeser, David, and Mark van der Laan. 2016. "The Highly Adaptive Lasso Estimator." *Proceedings of the ... International Conference on Data Science and Advanced Analytics. IEEE International Conference on Data Science and Advanced Analytics* 2016 (December): 689–96.

Coyle, Jeremy R., Nima S. Hejazi, Ivana Malenica, Rachael Phillips, and Oleg Sofrygin. 2021. *sl3: Modern Pipelines for Machine Learning and Super Learning* (version 1.4.2). https://doi.org/10.5281/zenodo.1342293.

Kennedy, C. 2017. "Guide to SuperLearner." https://cran.r-project.org/web/packages/SuperLearner/vignettes/Guide-to-SuperLearner.html.

Laan, Mark J. van der, Eric C. Polley, and Alan E. Hubbard. 2007. "Super Learner." *Statistical Applications in Genetics and Molecular Biology* 6 (1). https://doi.org/10.2202/1544-6115.1309.

LeDell, Erin, and Sebastien Poirier. 2020. "H2o Automl: Scalable Automatic Machine Learning." In *Proceedings of the AutoML Workshop at ICML*. Vol. 2020. automl.org. https://www.automl.org/wp-content/uploads/2020/07/AutoML_2020_paper_61.pdf.

Naimi, Ashley I., and Laura B. Balzer. 2018. "Stacked Generalization: An Introduction to Super Learning." *European Journal of Epidemiology* 33 (5): 459–64.

Polley, Eric C., and Mark J. van der Laan. 2010. "Super Learner In Prediction," U.C. Berkeley Division of Biostatistics Working Paper Series, . https://biostats.bepress.com/ucbbiostat/paper266/.

Polley, Eric C., Erin LeDell, Chris J. Kennedy, Sam Lendle, and Mark J. van der Laan. 2019. "Package 'SuperLearner.'" CRAN. https://CRAN.R-project.org/package=SuperLearner.

Polley, Eric C., Sherri Rose, and Mark J. van der Laan. 2011. "Super Learning." *Targeted Learning*. https://doi.org/10.1007/978-1-4419-9782-1_3.

Van Der Laan, Mark J., and Sandrine Dudoit. 2003. "Unified Cross-Validation Methodology for Selection among Estimators and a General Cross-Validated Adaptive Epsilon-Net Estimator: Finite Sample Oracle Inequalities and Examples." https://biostats.bepress.com/ucbbiostat/paper130/.

Wolpert, David H. 1992. "Stacked Generalization." *Neural Networks: The Official Journal of the International Neural Network Society* 5 (2): 241–59.

© 2022 Nock MK et al. *JAMA Network Open.*
